# Nutrient Composition and In Vitro Fermentation Characteristics of Sorghum Depending on Variety and Year of Cultivation in Northern Italy

**DOI:** 10.3390/foods11203255

**Published:** 2022-10-18

**Authors:** Ahmed Osman, Amr Abd El-Wahab, Marwa Fawzy Elmetwaly Ahmed, Magdalena Buschmann, Christian Visscher, Clara Berenike Hartung, Jan Berend Lingens

**Affiliations:** 1 Institute for Animal Nutrition, University of Veterinary Medicine Hannover, Foundation, Bischofsholer Damm 15, 30173 Hannover, Germany; 2 Department of Nutrition and Nutritional Deficiency Diseases, Faculty of Veterinary Medicine, Mansoura University, Mansoura 35516, Egypt; 3 Department of Hygiene and Zoonoses, Faculty of Veterinary Medicine, Mansoura University, Mansoura 35516, Egypt; 4 KWS LOCHOW GmbH, Grimsehlstraße 31, 37574 Einbeck, Germany

**Keywords:** sorghum, regions, nutrition, viscosity, in vitro fermentation

## Abstract

Sorghum is the fifth most produced cereal in the world and is a source of nutrients and bioactive compounds for the human diet. This study examined the nutrient composition and in vitro fermentation characteristics of sorghum varieties grown in 2020 and 2021 (*n* = 15 × 3 × 2) across three locations in the north of Italy (Bologna, Padova, and Rovigo). In 2020, the crude protein content of sorghum was significantly higher in the region of Padova than in the region of Bologna (124 vs. 95.5 g/kg dry matter). However, crude fat, sugar, and gross energy levels showed no significant differences among the different regions in 2020. In 2021, the levels of crude protein, crude fat, sugar, and gross energy had no significant differences among different sorghum varieties harvested from the three regions. Significant differences in some mineral contents were found among the samples particularly in manganese and zinc in both years. After 24 h of fermentation of two different sorghum hybrids (hybrids 1 and 2 of both years harvested in Bologna, *n* = 4 × 2 × 2), the pH value was significantly higher in hybrid 1 of year 2021 (3.98) than in the other fermented samples (range: 3.71–3.88). The sorghum harvested from the region of Bologna had a significantly higher viscosity value (1.22 mPa·s) compared to other regions (1.8–1.10 mPa·s) in 2021 only. The results show that the nutritional value and viscosity of different sorghum varieties could differ depending on the location and year of cultivation.

## 1. Introduction

Sorghum is a highly drought-tolerant field crop. It has low water requirements, and therefore is widely used as an important grain in many regions of the world [1]. This is reflected by the cultivated area on a global scale. Sorghum is the fifth most frequently grown grain worldwide after wheat, maize, rice, and barley [1,2,3] and has many applications. With an increasing world population and decreasing water supplies, sorghum represents an important crop for future human use. Worldwide, more than 35% of sorghum is grown directly for human consumption. The rest is used primarily for animal food/feed and industrial products [3,4]. Climate change effects immensely disturb the global agricultural systems by reducing crop production. Weather changes such as high-temperature, droughts, and flooding adversely affect the production of crops, posing a threat to ecosystem resilience [5,6]. Sorghum, ‘the camel of cereals’, is a climate-resilient food crop that is less sensitive to climatic changes and thus is considered as an important grain in many parts of the world [7]. 

All characters of grain quality, size, and composition as well as germination ability could be affected by environmental conditions. Several studies have proven the effect of the environment on grain composition and many of them have reported year to year variability and region–year interaction for grain quality traits [8,9,10]. The temperate and tropical regions are both suitable for the growth of sorghum. Soils with a pH between 5.5 and 8.5 can be used for the growth of sorghum. The minimum temperature requirement for germination is 7–10 °C; however, more than 80% of the sorghum seeds can germinate at 15 °C. The optimum temperature requirement for sorghum growth is 27–30 °C [11]. Sorghum grain color can vary substantially. It can appear white, yellow, red, brown, or black [12,13]. Starch is the major component of the sorghum grain and total starch levels in the grain can vary widely from 720 to 775 g/kg dry matter (DM) [13]. On average, sorghum contains 700 g/kg DM starch, thus starch is the main component for sorghum [14]. The protein content varies between 100–130 g/kg DM due to differences in genotypes, environments, crop management practices, and analytical methods according to different studies [15,16,17]. In addition to phytate, sorghum can also contain high amounts of tannins, which may negatively influence its utilization within the gastrointestinal tract [18]. However, European sorghum varieties have been cultivated to be low in tannins over the last 30 years. In fact, it is not possible to register new sorghum varieties unless their tannin concentration is lower than 30 g/kg [19,20]. Further knowledge about the nutrient composition and availability of regionally grown sorghum could be used to improve economic and ecologic sustainability in human food and animal feed [21,22]. 

The nutrient composition of sorghum indicates that it is a good source of vitamins and minerals including trace elements, particularly iron and zinc, except calcium. Sorghum grains contain minerals such as phosphorus, potassium, and magnesium in varying quantities [13]. Deficiencies in iron, iodine, vitamin A, and zinc are still a major public health problem in developing countries [23]. Sorghum is an excellent source of bioactive compounds that can promote benefits to human health. For example, about two billion people are deficient in zinc, and one billion suffer from iron-deficiency anemia [24,25,26], for which sorghum can compensate due to its high content of those minerals. It may also display positive effects on cardiovascular health and on cholesterol [27]. In addition, sorghum is often recommended as a safe food for celiac patients, who do not tolerate the protein gluten (both the gliadins and glutenins) of wheat, as it also provides a good basis for gluten-free breads and other baked products such as cakes and cookies (biscuits) as well as snacks and pasta [28].

Fermentation could play a significant role in improving the nutrient quality by drastically reducing the anti-nutritional factors to much safer levels than any other processing method tested [29]. Fermented sorghum may increase the nutritional value of food and feed [30,31]. Briefly, complex dietary compounds are broken down to simpler ones via a dynamic process involving microorganisms and substrates called ‘fermentation’ [32,33]. Furthermore, by using water and some microorganisms, the solid material (complete food or grain) can also be fermented [34,35]. Sorghum, for example, is considered suitable for fermentation due to its high starch content, which is a good carbohydrate source for fermentation. Studies on humans have shown that the digestibility of a crop as well as its nutritive value can be increased by applying proper fermentation [36,37].

Viscosity is an important and relevant attribute in liquid and semisolid food product categories [38]. Sorghum products are important foods with low viscosity and high energy density [39,40]. On the other hand, a low viscosity food contains a higher food quality [41]. The aim of this experiment was to evaluate the chemical/nutritional characteristics, amino acid values, viscosity, and in vitro fermentation characteristics of ground sorghum, analyzed from 90 samples of different hybrids received from three different locations in northern Italy (Bologna, Padova, and Rovigo) from the years 2020 and 2021. As far as we know, currently no information is available on the nutritional characteristics of these hybrids, which lends an added value to this study. 

## 2. Materials and Methods

### 2.1. Experimental Design and Description of Samples

**Figure 1:**
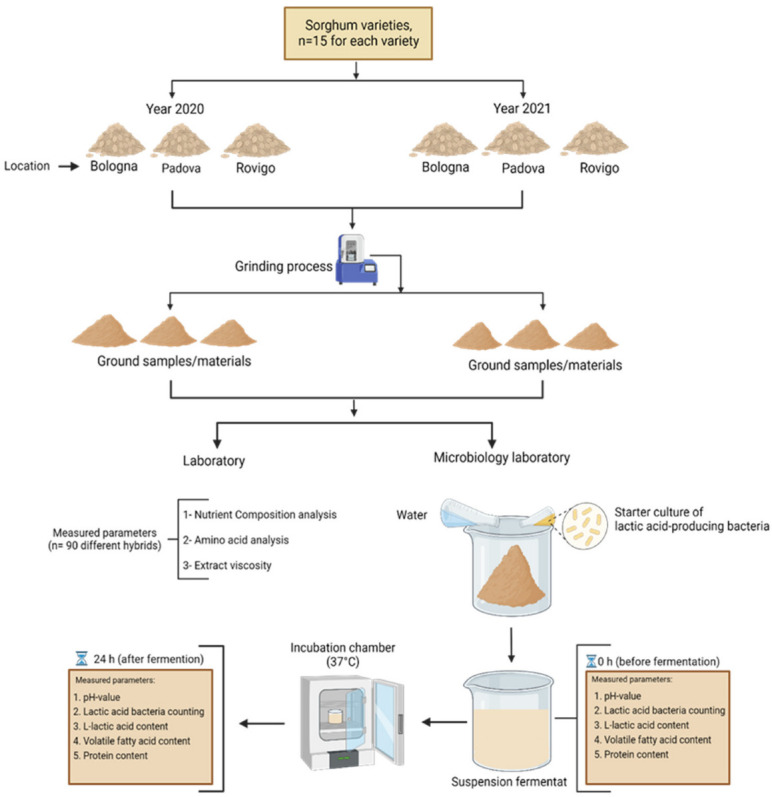
Experimental design for determining nutrient composition and for the fermentation process of sorghum (figure was created with BioRender.com on 25 May 2022).

This study included the collection and analysis of 90 samples of sorghum. Samples were harvested in three different locations of northern Italy, Padova (*n* = 30), Bologna (*n* = 30), and Rovigo (*n* = 30). Regarding soil quality, soil pH and soil fertilization, there were some differences in soil composition, for example, in the Bologna region (clay: 44%, silt: 46.5%, and sand: 9.5%; pH = 7.9; N = 30.3 mg/kg, P = 8.80 mg/kg, K = 147 mg/kg, Mg = 320 mg/kg, Fe = 35.4 mg/kg, Mn = 12.4 mg/kg, Cu = 7.9 mg/kg, Zn = 2.0 mg/kg); in the Padova region (clay: 45.9%, silt: 39.7% and sand: 14.4%; pH = 7.6; N = 32.3 mg/kg, P = 51.8 mg/kg, K = 403.0 mg/kg, Mg = 1600 mg/kg, Fe = 18.4 mg/kg, Mn = 2.4 mg/kg, Cu = 3.6 mg/kg, Zn = 2.2 mg/kg), and in the Rovigo region (clay: 26%, silt: 54.3%, and sand: 18.8%; pH = 7.2; N = 29.6 mg/kg, P = 11.6 mg/kg, K = 160 mg/kg, Mg = 340 mg/kg, Fe = 33.8 mg/kg, Mn = 13.2 mg/kg, Cu = 7.20 mg/kg, Zn = 3.20 mg/kg).The air temperatures were between 17–35 °C, sowing took place in late April/early May and harvest time was in September of the respective year in all locations. The average annual rainfall in these areas was 668 mm in 2020 and 553 mm in 2021. An overview of the experimental process can be found in Figure 1.

### 2.2. Grinding and Nutrient Composition 

The samples from both years were provided by KWS LOCHOW GmbH, Bergen, Germany. Samples were ground with Retsch ZM 200 Mill (Retsch GmbH, Haan, Germany) with sieve sizes of 1 mm (for fermentation, viscosity, and amino acid) or 0.5 mm (for nutrient composition). The ground sorghum was used for the analyses. The official methods of the Association of German Agricultural Analytic and Research Institutes (VDLUFA) were used for the analysis of the grains [42]. A hot air oven was used at 103 °C to determine the DM content. The crude ash content was estimated by using the muffle furnace at 600 °C. The Dumas incineration method (rapid Max Nexceed, Elementar, Analysensysteme GmbH, Langenselbold, Germany) was performed to estimate the total nitrogen content. Moreover, the crude fat content was measured by automatic hydrolysis (Hydrotherm) and automatic extraction (Soxtherm, C. Gerhardt GmbH & Co. KG, Köngiswinter, Germany). The samples were washed in diluted sulfuric acid and sodium hydroxide to determine the crude fiber content (Fibertec 2010, Foss Analytical A/S, Hillerød, Denmark). The starch content was measured by using a polarimetric method (Unipol, Schmidt + Haensch GmbH & Co., Berlin, Germany), while sugar content was estimated by titration according to Luff–Schoorl. To measure the mineral content, the atomic absorption spectrometry was used (ZEENIT 700, Analytik Jena GmbH, Jena, Germany). Finally, ion-exchange chromatography (AA analyzer LC 3000, Biotronik Wissenschaftliche Jena GmbH, Maintal, Germany) was used to analyze amino acid content.

### 2.3. Extract Viscosity

The extract viscosity values (*n* = 15 × 3 × 2) were calculated using a method described in [43], with a few modifications. About 5 g of ground grain and 20 mL of tap water were combined and stirred on a vortex mixer for 5 s. After 30 min at 38 °C, the samples were centrifuged for 10 min at 10,000× *g*. The viscosity was then determined using a viscosimeter (Model DVNext, DVNXLVCJG, Ametek Brookfield, MA, USA). In a viscometer measuring unit, about 600 µL was used in a S40 spindle rotating at 10 rpm and at a temperature of 26 °C. The specified value was recorded after one min.

### 2.4. In Vitro Fermentation

The fermentation was based on the method given by [35]. The sorghum varieties hybrids 1 and 2 were provided by KWS LOCHOW GmbH, Bergen, Germany. Four samples of hybrids 1 and 2 from both years, 2020 and 2021, harvested in Bologna, *n* = 4 × 2 × 2 were used. By using a 400 mL beaker, the ground sorghum grains (1 mm) were fermented in vitro. To obtain the aimed DM content of 37.5% for fermentation, the ratio of sorghum to water was controlled in the mixture before fermentation according to [35]. To avoid any adverse changes of fermentation, a freeze-dried, granulated starter culture (Schaumalac Feed Protect XP G, H. Wilhelm Schaumann GmbH, Pinneberg, Germany), consisting of 1k2079 *Lactobacillus plantarum*, 1k2103 *Pediococcus pentosaceus*, and 1k2082 *Lactococcus lactis* was added at the beginning of each fermentation process at a dose of 2 × 10^7^ colony-forming unit (CFU)/g ingredient. The beakers were incubated (Binder-Anaerobier Incubator, Fa. BINDER GmbH, Tuttlingen, Germany) anaerobically (CO_2_ = 10.0%, O_2_ = 0.2%, N = 89.8% and temperature = 37.0 °C) for 24 h.

#### 2.4.1. pH Values 

The pH values were analyzed directly according to [44] by using a calibrated glass electrode (HI 2211 pH/ORP meter, Hanna Instruments Deutschland GmbH, Vöhringen, Germany). 

#### 2.4.2. Lactic Acid Bacteria Counts

The count of lactic acid bacteria (LAB) was carried out in accordance with [35]. Samples (10 g) were taken at two distinct times from stomacher bags containing Schaumalac Feed Protect XPG once before fermentation (0 h) and after fermentation (24 h). Three different concentrations of Rogosa agar were performed, each with a volume of 100 µL, streaking in triplicate. The plates were incubated anaerobically (CO_2_ = 10.0%, O_2_ = 0.2%, N = 89.8%) at 37.0 °C for 48 h, then the colonies of LAB were counted and expressed in a Log_10_ CFU/g.

#### 2.4.3. L-Lactic Acid Content

The lactic acid content after fermentation was analyzed according to [44]. The perchloric acid (1 mol/L) was first added to the sample material and centrifuged to determine the l-lactate concentration. Potassium hydroxide solution was then added and mixed to supernatant (2 mL) until a pH range of 8–10 was reached. An enzymatic determination (L-lactic acid UV test, Roche Diagnostics GmbH, Mannheim, Germany) was performed after another centrifugation step. 

#### 2.4.4. Short Chain Fatty Acids

The concentration of short chain fatty acids (SCFA) in the fermented material was measured by gas chromatography (GC Shimadzu FID, Kyoto, Japan). The samples were mixed 1:5 with an internal standard (17% phosphoric acid with 4-methylvaleric acid) and subsequently stored at −20 °C. After thawing, samples were centrifuged for 15 min at 3000 rpm, diluted 1:4 with water and afterwards analyzed. Samples were subjected to gas chromatography with an oven temperature of 80 °C (maintained for 1 min) and heated to 225 °C at 8.5 °C/min (maintained for 5 min).

### 2.5. Statistical Analysis

The SAS^®^ Enterprise Guide^®^, version 9.3 of the Statistical Study System for Windows, was used to conduct the statistical analysis (SAS Institute Inc., Cary, NC, USA). Every parameter was examined for each sample (*n* = 15). In addition, the means and the standard deviations of the means (SD) were computed. A Ryan–Einot–Gabriel–Welsch test (basic ANOVA) was run to determine whether the data showed any significant differences under the presumption that they were normally distributed. Differences were deemed significant if their significant level was *p* < 0.05.

## 3. Results

### 3.1. Nutrient Composition

The analyzed nutrient composition of the different sorghum hybrids is shown in Table 1. The energy content of sorghum samples in both years (2020 and 2021) did not significantly differ and ranged between 18.0–18.1 MJ/kg DM. In 2020, the crude protein content of the sorghum samples was significantly higher in the region of Padova than in the region of Bologna (124 vs. 96.5 g/kg DM). Regarding levels of minerals in different sorghum varieties in 2020, significant differences were observed for phosphorus, magnesium, sodium, zinc, and manganese. In 2021 (Table 1), only significant differences were observed among the three regions in levels of copper, zinc, iron, and manganese.

#### Amino Acid Content

The levels of amino acids in sorghum harvested from the three different regions are shown in Table 2. In 2020, only levels of four amino acids (histidine, lysine, tyrosine, and glycine) showed significant differences among the three regions. Nevertheless, in 2021, levels of six amino acids (leucine, lysine, valine, asparagine, proline, and glycine) displayed significant differences among the three regions.

### 3.2. Viscosity

Table 3 shows the viscosity values (mPa·s) in sorghum from different locations and years. No significant differences were found in the viscosity values in 2020 among the different sorghum hybrids of the three regions (*p*-value = 0.3794). Nevertheless, in 2021, sorghum harvested in the region of Bologna had significantly the highest viscosity value (1.22 mPa·s) compared to the other regions (1.8–1.10 mPa·s).

### 3.3. In Vitro Fermentation Characteristics of Different Sorghum Hybrids and Years from Location Bologna

The fermentation characteristics of two sorghum varieties (hybrid 1 and hybrid 2) harvested in the region of Bologna in 2020 and 2021 are shown in Table 4. The pH values before fermentation were significantly different among the hybrids, whereas generally in 2020, the pH values were higher (6.31–6.32) than those in 2021 (6.15–6.25). Before fermentation, the LAB counts were significantly different only between hybrid 1 from 2020 and hybrid 2 from 2021 (3.82 vs. 3.39 log_10_ CFU/g).

The lactic acid content before fermentation of different sorghum hybrids did not differ significantly (range: 0.28–0.32 g/kg DM). The acetic acid content in unfermented hybrid 2 sorghum in 2020 was significantly higher (2.19 g/kg DM) than in the other unfermented samples (range: 1.42–1.59 g/kg DM). The propionic acid content in unfermented hybrid 2 sorghum in 2020 was significantly higher (0.07 g/kg DM) than in unfermented hybrid 1 sorghum from 2020/2021. Unfermented hybrid 1 harvested in 2021 had a significantly higher butyric acid content (0.13 g/kg DM) than in other samples (0.02 g/kg DM). Additionally, unfermented hybrid 1 harvested in 2021 had a significantly higher crude protein content (183 g/kg DM) compared to other samples (range: 123–147 g/kg DM).

After 24 h of fermentation of different sorghum hybrids, the pH value was significantly lower in hybrid 1 from 2020 (3.71) than in other fermented samples (range: 3.81–3.98). The acetic acid content in the fermented hybrid 1 from 2021 was significantly higher (3.62 g/kg DM) compared to other fermented sorghum samples (range: 1.87–3.16 g/kg DM). The propionic acid content was identical in all the fermented sorghum samples (0.11 g/kg DM). The butyric acid content in fermented sorghum samples from year 2021 was significantly higher than those samples from 2020. The fermented hybrid 1 harvested from 2021 had a significantly higher crude protein content (184 g/kg DM) than the other samples (range: 128–153 g/kg DM).

## 4. Discussion

In the current study, we compared the chemical composition of different varieties of sorghum grown in three regions in northern Italy. The development of novel sorghum hybrids with superior functional and nutraceutical qualities when cultivated in the Mediterranean area would encourage the use of sorghum for human consumption as a nutritious food in European nations. Additionally, because sorghum is a drought-tolerant plant that is extremely well adapted to environmental changes, it may encourage farmers in Europe to grow it [45]. It has been reported that the color of the pericarp of sorghum grain may vary due to both genotype and environmental factors [46,47,48]. 

Sorghum grain is a good source of energy, carbohydrates, some essential amino acids, minerals, and vitamins [45]. Earlier studies have shown that the proximate composition of sorghum carbohydrate (54.6–85.2%) and protein (6.2–14.9%) contents vary considerably [30]. In the current study, the composition profiles of sorghum varieties grown in northern Italy from the three different locations (Padova, Bologna, and Rovigo) for two years were similar overall, with slight differences in both protein and carbohydrate percentages. In 2020, with regard to amino acid composition in the three locations, four amino acids differed significantly. Nevertheless, in 2021, six amino acids from the whole amino acid profile differed significantly. The sorghum variety, soil and meteorological conditions, and the stage of plant maturity at harvest could all have an impact on the variations in amino acid contents between the three sorghum locations. 

The current study standardized the fertilization applied at the three sites, leaving the variations to the individual sites. Therefore, the sorghum variety, soil and/or weather conditions, and the level of plant maturity at harvest could all have an impact on the variations in the quantities of some trace elements between the three sorghum locations [49]. The presented results coincide with [50], who pointed out the need to conduct experimental investigations under local conditions by stating that the mineral levels in sorghum differed depending on genotypic and environmental effects.

In both years (2020 and 2021), the content of the major elements followed the sequence: potassium > magnesium > calcium > sodium in sorghum varieties from all three locations analyzed, with the primary mineral being potassium, followed by magnesium, which is consistent with the literature [51,52]. According to [49], the content of essential minerals followed the sequence: potassium> phosphorus > magnesium> sodium> calcium in the analyzed samples. Potassium and sodium content for the samples varied from 3.43 to 6.96 g kg^−1^ and 0.489 to 0.840 g kg^−1^, respectively. On the other hand, the sorghum hybrids differed significantly with respect to calcium and phosphorus, and their contents varied from 0.233 to 0.411 g kg^−1^ and from 2.15 to 2.96 g kg^−1^, respectively. A good diet should have a calcium:phosphorus ratio over 1.0 [53]. Since the sorghum hybrids recorded a low calcium:phosphorus ratio (about 0.14), a large consumption of sorghum flour should be accompanied with calcium supplementation to prevent mineral and osmotic imbalance [54]. With regard to the content of major elements, our study reported a potassium:sodium ratio with a great value (range: 44.8–56.3 in year 2020 and 137–142 in year 2021) for all sorghum varieties analyzed as mentioned by [55]. The sorghum variants had a higher magnesium content than is generally found in maize (on average, 0.47 g kg^−1^) and wheat flour (on average, 0.25 g kg^−1^) [56]. The most abundant trace elements were iron and zinc in all sorghum varieties analyzed, confirming the data reported in the literature [47,49,51]. These latter elements are crucial trace elements for human and animal nutrition, and an iron shortage, for instance, is a major global public health risk [57]. 

The growing sorghum production for human consumption in the United States and in Mediterranean nations points to the potential for this crop to provide wholesome nutrition [28,45]. Choosing sorghum varieties for production in Europe depends on identifying those with the highest levels of iron. Regarding the trace element contents, the results reported in the present study show high contents of both iron and zinc in all the sorghum samples. 

With regard to viscosity values for the sorghum samples of the three locations harvested, no significant differences were found for 2020. However, in 2021, significant differences among the locations could be shown, whereas samples cultivated in Bologna had the highest viscosity value. In humans, in recent years, high-viscosity foods have received great attention due to the health benefits they bring after ingestion. A number of studies have shown that high-viscosity foods can slow down both the eating rate and the gastric emptying rate, which imparts a stronger feeling of satiety compared with low-viscosity foods, leading to a reduction in food intake and a decrease in the risk of obesity [58]. In addition, the intake of high-viscosity foods can help reduce both blood glucose levels and insulin response after meals, thereby reducing the risk of diabetes [59]. Generally, in humans, a higher viscosity in a food that can induce a decrease in the emptying rate of the stomach and slow gastric emptying may cause postprandial hypoglycemia [60]. Despite the fact that there are many methods for converting sorghum into other food forms, fermentation is still one of the oldest and important because of the beneficial properties it provides in food [30]. However, the viscosity value of a grain and/or diet is a critical issue concerning monogastric animal health. It is well known that arabinoxylans (complex cell wall polysaccharides) enhance digesta viscosity [61], which might lead to an impairment in passage rate and absorption of nutrients [62,63]. Another drawback of viscosity is that it can retain water in the digestive tract, resulting in sticky excreta and increased moisture in the litter of poultry houses [64,65]. 

Generally, fermentation is the deliberate conversion/modification of a substrate into new products/forms through microbial processes that alter the initial substrate and produce metabolites [30]. These modifications affect the resultant products’ nutritional value, shelf life, texture, color, and flavor. Additionally, fermentation results in the creation of enzymes that cause the substrates to break down, enhancing the nutritional value [66]. It has also been documented in the literature that the synthesis of organic acids can result in changes to functional qualities (such emulsifying and oil- and water-binding ability) as well as a drop in pH and an increase in acidity [67,68,69].

According to our study, the measured pH value of the fermented grains (with minimal DM content) is consistent with readings from [70] and [71] after fermenting liquid milled wheat. At 24 °C, a recent study [70] found that the pH was 3.9 after 24 h and 3.7 after 48 h of incubation. A variety of enzymes, either bacterial or endogenous to the grains, may be activated as a result of the pH decreasing during fermentation as a result of the creation of organic acids (mostly lactic and acetic). Typically, sorghum fermentation alters and improves the nutritional value, flavor, shelf life, aroma, and structural characteristics. Similar to other cereals, sorghum undergoes fermentation, which modifies its natural metabolites and constituents, activates enzymes, lowers pH levels, increases metabolic activity, and stimulates microbial activity. As a result, anti-nutritional factors are reduced, contaminants are detoxified, and other contaminants are degraded [30,66,72]. 

For sorghum, the counts of LAB and lactic acid levels did not vary among groups after 24 h of fermentation. Fermented sorghum at 12, 24, and 48 h had pH values ranging from 6.27 to 6.51 for a DM concentration of 75% and from 3.77 to 3.93 for a DM content of 25%, respectively [35]. Generally, in sorghum, due to their high acidity tolerance and relative superiority in the use of starchy sorghum substrates, LAB are the most prevalent microorganisms during fermentation, with little evidence of yeasts and fungi [36]. Therefore, it is not unexpected that lactic acid fermentation, which is mostly performed by LAB, is the most prevalent kind of sorghum fermentation [30]. 

In the current study, the crude protein of fermented and unfermented sorghum were of a particular importance, just as in our earlier investigation [35], where sorghum was fermented for 24 or 48 h, and the protein content did not differ between groups with different DM concentrations (except between 25% DM and 50% DM after 24 h). Several studies reported an increase [69,73,74], while others observed a decrease [75,76] in protein and/or some amino acids upon fermentation. 

The majority of these impacts seem to be relative changes brought on by the loss of DM as a result of microbes hydrolyzing and metabolizing carbs and lipids as energy sources, rather than genuine alterations. Recently, the authors of [44] found that a controlled fermentation had no appreciable impact on the amount of crude protein and the distribution of amino acids in liquid feed. According to a recent study [33], the concentration effects throughout the fermentation process led to increases in the protein content of fermented feed ingredients, whereas other nutrients are fermented by the microorganisms. The protein increases that have been noticed are therefore proportional alterations. The fermentation of sorghum has historically been carried out on a small, domestic scale such as *niselo* and *umqombothi* in South Africa. By fermenting the sorghum with a particular commercial LAB strain, a non-alcoholic beverage from Ting called *Motoho* was created [77]. The West African *kunu* and Ugandan *bushera*, the Kenyan gruel *uji*, and the Nigerian *ogibaba* are other notable and important fermented sorghum products [30,78]. 

Lactic acid is produced by LAB when they are combined with water, which lowers the pH of the mixture [70]. Thus, we intended to employ the DM concentration of sorghum that was most appropriate and/or promising for the current investigation (37.5% DM). According to a recent study [79], after 24 h, the lactic acid content of fermented sorghum with the addition of LAB was significantly higher than that of the control fermented sorghum (without LAB) (303 vs. 13.2 mmol/L). Furthermore, the authors of [32] found that the fermentation of red and white sorghum for 24 h resulted in the production of 312 and 314 mmol/L of lactic acid, respectively. However, there was a difference in the amounts of acetic acid in the control fermented sorghum (without LAB) and the fermented sorghum (with LAB) (5.42 and 10.6 mmol/L, respectively). The authors of a recent study [35] found that the DM content of 25% resulted in the maximum L-lactic acid level (22.2 g/kg and 24.4 g/kg DM) of fermented sorghum after either 24 or 48 h, respectively. The fermentation technique was used by the authors to enhance some physicochemical and nutritional characteristics of rye and sorghum (mixed with different ratios of water), while, the lowest L-lactic acid concentration was found in the fermented sorghum, which had a 75% DM content, after 24 or 48 h (0.13 and 0.19 g/kg DM, respectively). Overall, the hybrid type did not have an impact on the LAB count or L-lactic acid content among the fermented sorghum hybrids in the current investigation. So, any of the sorghum hybrids utilized in this investigation can be fermented well.

## 5. Conclusions

It is generally known that sorghum is a genetically diverse crop; this variety includes nutrient levels and composition, which leads to the expression of nutritional value in sorghum grain. Thus, sorghum has been studied including the role of its nutrients present in types of sorghum that vary depending on year and location of cultivation as well as fermentation. The present study supports the continued strategy of evaluating sorghum for additional nutritional properties such as carbohydrate (energy) content, protein and/or amino acids levels and minerals. Furthermore, the current study showed that sorghum growing in different cultivated locations can vary in overall nutrient composition. Overall, the sorghum variety, soil and meteorological conditions, and the stage of plant maturity at harvest could all have an impact on the variations in amino acid content between the three sorghum locations. Fermentation of different hybrids of sorghum in the same cultivated region varied mostly in pH value and protein content without any impact on the LAB count or L-lactic acid content.

## Figures and Tables

**Table 1:** Chemical composition of sorghum samples from different locations and years (*n* = 15 per location and year; mean ± SD).

Year	Parameter	Unit	Bologna	Padova	Rovigo	*p*-Value
2020	Dry matter	(g/kg fresh basis)	897 ± 7.18	899 ± 8.10	895 ± 7.80	0.7441
	Crude ash	(g/kg DM)	16.4 ± 1.06 ^b^	18.7 ± 2.35 ^a^	16.2 ± 1.47 ^b^	0.0002
	Crude protein		96.5 ± 21.1 ^b^	124 ± 29.3 ^a^	109 ± 14.6 ^ab^	0.0044
	Crude fat		42.7 ± 2.59	42.9 ± 3.10	42.1 ± 3.20	0.7563
	Crude fibre		30.3 ± 2.10	32.3 ± 2.03	32.1± 3.75	0.1035
	NfE ^1^		814 ± 22.3 ^a^	782 ± 30.9 ^b^	800 ± 16.6 ^a^	0.0026
	Starch		775 ± 20.4 ^a^	725 ± 17.2 ^c^	752 ± 11.9 ^b^	˂0.0001
	Sugar		11.5 ± 1.41	11.7 ± 1.38	11.1 ± 2.67	0.7041
	Gross energy ^2^	(MJ/kg DM)	18.1 ± 0.33	18.0 ± 0.73	18.1 ± 0.43	0.6736
	Calcium	(g/kg DM)	0.11 ± 0.00	0.11 ± 0.00	0.11 ± 0.00	0.3371
	Phosphorus		3.11 ± 0.23 ^ab^	3.43 ± 0.49 ^a^	3.00 ± 0.64 ^b^	0.0503
	Magnesium		1.25 ± 0.09 ^b^	1.45 ± 0.17 ^a^	1.36 ± 0.15 ^a^	0.0030
	Sodium		0.09 ± 0.07 ^a^	0.07 ± 0.01 ^b^	0.07 ± 0.08 ^b^	˂0.0001
	Potassium		4.03 ± 0.27	3.80 ± 0.25	3.94 ± 0.41	0.1127
	Copper	(mg/kg DM)	3.50 ± 1.11	3.07 ± 0.51	3.31 ± 1.06	0.4644
	Zinc		18.0 ± 2.36 ^b^	23.2 ± 3.10 ^a^	21.2 ± 3.17 ^a^	˂0.0001
	Iron		35.3 ± 6.92	39.4 ± 6.60	37.1 ± 4.30	0.1857
	Manganese		5.65 ± 0.09 ^a^	5.62 ± 0.07 ^b^	5.62 ± 0.04 ^b^	˂0.0001
2021	Dry matter	(g/kg fresh basis)	912 ± 2.01	909 ± 2.22	910 ± 3.77	0.7351
	Crude ash	(g/kg DM)	18.0 ± 2.65	17.3 ± 0.71	17.2 ± 4.02	0.8554
	Crude protein		116 ± 7.77	110 ± 10.2	117 ± 16.5	0.1228
	Crude fat		45.0 ± 4.39	44.6 ± 3.11	41.2 ± 11.2	0.4114
	Crude fibre		27.2 ± 3.01	26.3 ± 2.10	26.4 ± 3.50	0.4650
	NfE^1^		794 ± 12.7	804 ± 11.2	798 ± 20.9	0.2127
	Starch		734 ± 12.6	746 ± 13.3	738 ± 22.1	0.1410
	Sugar		13.1 ± 1.34	12.0 ± 1.62	13.1 ± 1.02	0.0284
	Gross energy ^2^	(MJ/kg DM)	18.0 ± 0.22	18.0 ± 0.15	18.0 ± 0.50	0.7799
	Calcium	(g/kg DM)	0.11 ± 0.00	0.11 ± 0.00	0.18 ± 0.26	0.3787
	Phosphorus		3.43 ± 0.37	3.52 ± 0.70	3.36 ± 0.66	0.5716
	Magnesium		1.52 ± 0.36	1.46 ± 0.08	1.40 ± 0.21	0.3781
	Sodium		0.03 ± 0.00	0.03 ± 0.00	0.03 ± 0.00	0.1632
	Potassium		4.25 ± 0.32	4.11 ± 0.37	4.10 ± 0.00	0.1709
	Copper	(mg/kg DM)	2.80 ± 0.01 ^b^	2.81 ± 0.06 ^b^	5.33 ± 1.43 ^a^	˂0.0001
	Zinc		22.2 ± 2.18 ^b^	19.4 ± 2.20 ^c^	24.5 ± 2.70 ^a^	˂0.0001
	Iron		45.9 ± 6.47 ^a^	32.5 ± 5.22 ^b^	35.1 ± 6.02 ^b^	˂0.0001
	Manganese		5.54 ± 0.01 ^c^	6.78 ± 1.63 ^b^	8.86 ± 2.23 ^a^	˂0.0001

^1^ NfE: nitrogen-free extract = 1000 − (crude ash + crude protein + crude fat + crude fiber); ^2^ Gross energy = (0.239 × crude. protein + 0.0398 × crude fat + 0.0201 × crude fiber + 0.0175 × NfE); ^a,b,c^ Means in a row with different superscripts differ significantly (*p* < 0.05). SD: standard deviation of means.

**Table 2:** Amino acid content (g per 100 g protein) of sorghum from different locations and years (*n* = 15 per location and year; mean ± SD).

Year	Parameter	Bologna	Padova	Rovigo	*p*-Value
2020	Arginine	4.12 ± 0.36	3.73 ± 0.89	3.74 ± 0.24	0.1187
	Histidine	2.31 ± 0.15 ^a^	2.11 ± 0.14 ^b^	2.18 ± 0.14 ^b^	0.0024
	Isoleucine	3.24 ± 0.20	3.70 ± 0.25	3.78 ± 0.22	0.6550
	Leucine	12.1 ± 0.75	12.4 ± 0.90	12.1 ± 0.93	0.5297
	Lysine	2.47 ± 0.34 ^a^	2.00 ± 0.19 ^b^	2.32 ± 0.24 ^a^	˂0.0001
	Methionine	2.02 ± 0.51	1.96 ± 0.33	2.50 ± 0.50	0.3424
	Phenylalanine	4.93 ± 0.29	4.92 ± 0.34	4.83 ± 0.24	0.6100
	Threonine	3.10 ± 0.15	3.05 ± 0.24	3.14 ± 0.23	0.4756
	Valine	4.98 ± 0.23	4.75 ± 0.33	4.92 ± 0.30	0.1029
	Alanine	8.58 ± 0.69	8.67 ± 0.58	8.43 ± 0.47	0.5355
	Aspartic acid	6.74 ± 0.46	6.51 ± 0.45	6.42 ± 0.28	0.1065
	Cysteine	2.56 ± 0.69	2.04 ± 0.51	2.34 ± 0.63	0.0808
	Glutamic acid	19.6 ± 2.53	21.1 ± 2.13	19.7 ± 1.45	0.1068
	Proline	7.59 ± 0.38	8.09 ± 0.91	7.60 ± 0.47	0.0577
	Serine	4.31 ± 0.25	4.20 ± 0.27	4.31 ± 0.24	0.0749
	Tyrosine	3.60 ± 0.17 ^a^	3.39 ± 0.43 ^b^	3.54 ± 0.21 ^ab^	0.0411
	Glycine	3.51 ± 0.38 ^a^	3.00 ± 0.27 ^b^	3.22 ± 0.26 ^b^	0.0002
2021	Arginine	3.69 ± 0.26	3.84 ± 0.25	3.81 ± 0.28	0.2779
	Histidine	2.18 ± 0.10	2.26 ± 0.23	2.21 ± 0.16	0.5221
	Isoleucine	3.92 ± 0.19	3.87 ± 0.08	3.96 ± 0.24	0.4580
	Leucine	12.4 ± 0.42 ^b^	12.0 ± 0.32 ^b^	13.0 ± 0.90 ^a^	0.0002
	Lysine	2.13 ± 0.15 ^b^	2.32 ± 0.17 ^a^	2.30 ± 0.21 ^a^	0.0118
	Methionine	2.16 ± 0.23	2.07 ± 0.21	2.16 ± 0.33	0.5759
	Phenylalanine	4.89 ± 0.13	5.52 ± 0.75	5.00 ± 0.26	0.1641
	Threonine	3.18 ± 0.19	3.39 ± 0.11	3.20 ± 0.33	0.0407
	Valine	4.86 ± 0.10 ^b^	5.02 ± 0.12 ^a^	5.05 ± 0.28 ^a^	0.0262
	Alanine	8.85 ± 0.26	8.76 ± 0.20	9.05 ± 0.61	0.1583
	Aspartic acid	6.38 ± 0.19 ^c^	6.63 ± 0.13 ^b^	6.95 ± 0.35 ^a^	˂0.0001
	Cysteine	2.14 ± 0.37	2.00 ± 0.17	1.96 ± 0.19	0.1701
	Glutamic acid	20.8 ± 0.56	21.2 ± 0.55	21.4 ± 1.47	0.1640
	Proline	7.71 ± 0.20 ^b^	7.90 ± 0.29 ^b^	8.23 ± 0.23 ^a^	0.0015
	Serine	4.27 ± 0.10	4.39 ± 0.13	4.31 ± 0.24	0.1845
	Tyrosine	3.50 ± 0.11	3.44 ± 0.10	3.39 ± 0.24	0.1828
	Glycine	3.11 ± 0.14 ^b^	3.33 ± 0.23 ^a^	3.24 ± 0.28 ^ab^	0.0413

^a,b,c^ Means in a row with different superscripts differ significantly (*p* < 0.05) SD: standard deviation of means.

**Table 3:** Viscosity values (mPa·s) in sorghum from different locations and years (*n* = 15 per location and year; mean ± SD).

Year	Bologna	Padova	Rovigo	*p*-Value
2020	1.33± 0.33	1.30 ± 0.31	1.20 ± 0.13	0.3794
2021	1.22 ± 0.20 ^a^	1.08 ± 0.07 ^b^	1.10 ± 0.08 ^b^	0.0100

^a,b^ Means in a row with different superscripts differ significantly (*p* < 0.05). SD: standard deviation of means.

**Table 4:** pH values, count of lactic acid bacteria (LAB, log_10_ CFU/g), L-lactic acid content (g/kg DM), SCFA concentrations (g/kg DM), and protein content (g/kg DM) in the fermented samples (37.5% DM-content) before (0 h) and after (24 h) fermentation (*n* = 4; mean ± SD).

Parameters	Time, h	Hybrid/Year				
		Hybrid1 2020	Hybrid2 2020	Hybrid1 2021	Hybrid2 2021	*p*-Value
pH-value	0	6.32 ± 0.03 ^a^	6.31 ± 0.01 ^a^	6.15 ± 0.03 ^c^	6.25 ± 0.00 ^b^	˂0.0001
Count of LAB		3.82 ± 0.07 ^a^	3.71 ± 0.16 ^ab^	3.68 ± 0.17 ^ab^	3.39 ± 0.25 ^b^	0.0281
L-lactic acid		0.32 ± 0.03	0.31 ± 0.03	0.28 ± 0.01	0.29 ± 0.01	0.0861
Acetic acid		1.59 ± 0.33 ^b^	2.19 ± 0.28 ^a^	1.42 ± 0.03 ^b^	1.57 ± 0.15 ^b^	0.0022
Propionic acid		0.04 ± 0.00 ^b^	0.07 ± 0.01 ^a^	0.04 ± 0.01 ^b^	0.06 ± 0.00 ^ab^	0.0009
Butyric acid		0.02 ± 0.01 ^b^	0.02 ± 0.01 ^b^	0.13 ± 0.08 ^a^	0.02 ± 0.00 ^b^	0.0027
Crude protein		147 ± 2.00 ^b^	123 ± 2.00 ^c^	183 ± 2.65 ^a^	147 ± 3.68 ^b^	˂0.0001
pH-value	24	3.71 ± 0.01 ^b^	3.88 ± 0.01 ^b^	3.98 ± 0.02 ^a^	3.81 ± 0.03 ^c^	˂0.0001
Count of LAB		9.39 ± 0.16	9.53 ±0.34	9.22 ± 0.04	9.22 ± 0.12	0.1354
L-lactic acid		16.6 ± 1.60	16.7 ± 1.51	16.1 ± 1.05	16.9 ± 1.18	0.8203
Acetic acid		2.91 ± 0.19 ^a^	1.87 ± 0.20 ^b^	3.62 ± 1.01 ^a^	3.16 ± 0.45 ^a^	0.0063
Propionic acid		0.11 ± 0.07	0.11 ± 0.04	0.11 ± 0.02	0.11 ± 0.03	0.9988
Butyric acid		0.49 ± 0.09 ^b^	0.20 ± 0.11 ^b^	2.19 ± 0.75 ^a^	1.72 ± 0.50 ^a^	0.0001
Crude protein		150 ± 0.60 ^b^	128 ± 2.47 ^c^	184 ± 2.99 ^a^	153 ± 0.97 ^b^	˂0.0001

^a,b,c^ Means within a row without a common superscript differ (*p* < 0.05), SD: standard deviation of means.

## Data Availability

The original contributions generated for the study are included in the article; further inquiries can be directed to the corresponding author.
